# Compound Fractures, Amputations, &c.

**Published:** 1849-12

**Authors:** 


*Compound Fractures, Amputations, &c.*—In the New York Journal of Medicine for November, we find an interesting paper, with statistics, by Dr. John O. Stone, of the city of New York, on amputations and compound fractures. The suppression of the Astor Place riot, by the military, presented an interesting field of observation to Dr. Stone, and he has improved it. The statistics used by him are necessarily imperfect, but tend to urge the surgeon to greater efforts at saving limbs, than are usually made. The knife is often resorted to very unnecessarily, and we hail, with satisfaction, all efforts that have a tendency to teach caution in its use.

From a portion of his statistics, Dr. Stone draws the following conclusions:

1st. That primary amputations of the upper extremities are equally successful and to be preferred both in military and civil surgery.

2d. That, in military surgery, primary amputations of the lower extremities are twice as successful as secondary.

3d. That in civil surgery, (i. e. in the American hospitals,) it is immaterial whether primary or secondary amputations of the lower extremities are resorted to.

4th. That secondary amputations of the upper extremities in civil surgery, are eight per cent. less fatal than in military surgery.

5th. That secondary amputations in civil surgery, are twelve per. cent. less fatal than in military surgery.

In the above conclusions, all that is said of civil surgery embraces only the hospitals of Boston, New York and Philadelphia. But by adding together all the information we have been able to obtain with regard to amputation of the lower extremities in civil hospitals, and comparing it with the result obtained in military hospitals, we have

| | *Primary.* | | *Secondary.* | |
|---|---|---|---|---|
| American Hospitals, | 45 cases, | 17 deaths. | 44 cases, | 3 deaths. |
| North Liverpool Hosp. | 23 " | 20 " | 9 " | 4 " |
| University Coll. Hosp. | 5 " | 2 " | 42 " | 5 " |
| | 73 " | 39 " | 95 " | 12 " |

A mortality in primary amputations of lower extremities in civil practice of $53\frac{1}{2}$ per cent., and of $12\frac{2}{3}$ per cent. in secondary amputations. This gives us 32 per cent. in favor of primary amputations in military service compared with civil practice, and 36 per cent. in favor of secondary amputations of lower extremities in civil surgery. We also find, that there is a difference of 41 per cent. in favor of secondary amputations in civil surgery over primary, and a difference of 9 per cent. in favor of secondary amputations of civil practice, compared with the primary amputations of military surgery.

We may therefore conclude, *that, in military practice, primary amputations are to be preferred; that, in civil practice, secondary amputations are to be preferred; and that the secondary amputations of civil surgery are more successful than the primary of military surgery.*

This result is important, and if true, is subversive of the commonly entertained notions upon the subject of amputations, and must lead to a change of practice.

After paying some well-merited compliments to M. Bauden, "Surgeon of Val de Grace, and formerly with the army in Algiers," Dr. Stone quotes the following rules of practice in compound fractures, and particular injuries of the joints, from M. Bauden:

1st. Make no débridement, (freeing of parts by incisions.)

2d. Endeavor to make a complicated wound simple, by removing splinters of bone.

3d. Apply cold water and even ice.

4th. Endeavor to keep the inflammation strictly local, and thus prevent its extending to the viscera.

5th. When the upper extremity is comminuted, *remove the splinters, and make such incisions of osseous matter as may seem advantageous, and reserve secondary amputation as a last resource.*

6th. When the femur is shattered and splintered, amputate at once. When tibia or fibula, remove the splinters and give the limb a chance. When both bones are fractured, as a general rule, amputate immediately.

By an adherence to these judicious rules, especially the third one, M. Bauden says he has "saved two cases out of three, where the knee joint was opened. Of fourteen primary operations, three died. Out of six secondary, six died. *Twenty-five soldiers, who were shot in the limbs, and from whom he immediately removed all the splinters, all recovered.*"

Dr. Stone produces abundant proof to show that bones injured by gunshot wounds require large openings for the exit of pus. The philosophic Hennan is quoted for the observation that wounds of the knee joint, *with large openings*, are more easily and successfully managed than small wounds. Dr. Gibb furnishes statistics in proof of this fact. These cases occurred in Paris during the insurrection of June, 1848. There were twelve cases of compound fracture of the thigh, and six of them died, a result more successful than is obtained by amputations in such cases. The most gratifying point in Dr. Gibb's reports is the successful treatment of wounds of the knee joint. Five of the cases reported by him were wounds of the knee joint—"the most dangerous kind of wound"—of these three recovered without anchylosis, or bad symptom ; one recovered, with anchylosis.

These are certainly gratifying results, so far as a comparison is made between this mode of treatment, and the results of amputation. In connection with these results, Dr. Stone speaks highly of the rules of practice in such cases, which govern Dr. William J. Walker, of Boston. These rules were presented by Dr. Walker, in his annual address before the Massachusetts Medical Society, in 1845. These rules are, says Dr. Stone, "proved to be correct, by cases from his own practice, and by an array of cases by such men as Martiniere, Cannac, Boucher, etc., *until their very number imposes upon us*, and we are forced to admit the simple but certain points of practice which he inculcates." We can find space for but two of these rules, and we do so to show the extent of error that prevails, and to make some comment upon the whole of this surgery. The two first rules laid down by Dr. Walker are :

1st. That all membranous or tendinous structures that obstruct the removal of foreign bodies, or unduly confine or strangulate the soft parts, when swollen by inflammation, or which, by their presence, would be likely to prevent the free discharge of matter, should be freely divided.

2d. That such dependent orifices should be preserved or counter openings made, as will, when aided by position and dressings, secure the free discharge of matter which might otherwise stagnate within the wound.

It seems not to have entered into the conceptions of Drs. Walker and Stone, while contending for a higher and purer surgery than the amputating knife presents, that there was a height far above their own. For the kind of surgery that theypractice and inculcate, their rules are excellent, but there is a kind of surgical science that makes these rules an utter abomination. Instead of all this care about large openings, dividing membranous and tendinous structures, preserving dependent orifices, or making counter openings for the exit of matter, would it not manifest a higher degree of skill to prevent the formation of matter, and thus render these barbarous measures for its discharge, unnecessary ? The powers of the bandage are so obvious, so simple and successful in all these cases ; they are so superlatively beyond all other means in their successful results, that we are at a loss to imagine how any other rules than those that belong to the proper application of the simple roller, can, by any possibility, find a lodgment in the mind of the surgeon. We have seen the most formidable injuries to joints, tendons, and muscles ; comminuted, compound and simple fractures, invariably yield to the influence of the bandage, and many of them must have had, judging from observation of other methods, a serious termination under any other treatment. Under the influence of the roller, we have seen formidable gunshot wounds assume the aspect of ordinary incisions, and heal kindly in a space of time usually required for the sloughing process to complete itself. The formidable sloughing, immoderate flow of pus, fistulous formations, long continued illness and confinement are all absent under this treatment. Upwards of twenty years ago, we saw a case of injury that would have required amputation, and which must have ended in death, but for the bandage. Professor Dudley has recorded this case, and we cheerfully bear testimony to the accuracy of his details, for we saw the child dragged from its perilous place, and was incredulous as to the possibility of her recovery. Prof. Dudley says:

A little girl, twelve years of age, playing in a horse-mill, was caught by the knee, between the principal wheel and the walls of the building, and was carried round to a point where the approximation was so close as to stop the machinery propelled by four horses. After remaining between five and ten minutes in this situation, by the aid of levers and wedges the limb was disengaged. On examination, a few minutes after, I found all the integument, cellular substance, and most of the fascia protecting the muscles and tendons in the ham, for eight inches up and down the limb, were removed, and for about four inches around. The tendons of the biceps, semitendinosus and semimembranosus were exposed for at least four inches, and the head and upper portion of the gastrocnemius to a still greater distance; most of the bursæ being scraped off, thus leaving the tendons ragged and bare. The head of the fibula was completely denuded. A small portion of integument, rolled up like a cord, was left on the outer portion of this extensive wound. On the inner and front portion of the limb and a little above the knee, a spot of some magnitude, of a livid appearance, indicated the opposite seat of pressure and violence.

The bandage was immediately applied, from the toes to the hip, for the purpose of preventing all swelling and inflammation, and to keep the limb extended during the cure. When the dressing was applied the limb was nearly divested for the time of sensibility.

On the fifth day from the accident, the dressings were removed for the first time, when the integument that was killed all came away, leaving a suppurating surface without either inflammation or swelling. On the twelfth day, and third dressing, by the process of sloughing, the whole of the lacerated fascia came away and left the entire surface of the wound healthy and suppurating. The flexor tendons were so extensively exposed that fears were entertained that they might slough, especially that of the biceps, the whole of which was exposed. All fears on that head were speedily dissipated by the appearance of granulations on all the tendinous surface. But from causes not known a fever now supervened, which was only subdued after a patient and unremitting effort of fifteen days. During the continuance of the fever, the patient lost as much by ulceration as the sore had gained previously by the granulating process, so that at the expiration of the first four weeks, the wound was as large as at first, with a most obstinate disposition to contraction in the flexor muscles, created by some inflammation which had taken place. For it must be understood that from the loss of soft parts, the irregular conformation of the joint, and the impossibility of making complete, equable, and efficient pressure upon all points, the disposition to inflammatory action could not be entirely controlled. Under the persevering use of the bandage for another month, however, no medicated application being made to the wound, she was so far restored as to render farther attention on my part unnecessary. The occasional use of a splint, in aid of the bandage, was resorted to in order to counteract the flexor muscles of the limb.

Great apprehensions had been entertained for the preservation of the joint, because of the inflammation and the protracted nature of the case ; but before the expiration of the third month, she was enabled to walk the streets with but partial stiffness in the joint, and that was temporary in character.

The frightful extent of the wound, the great loss of soft parts, and the contusion resulting from pressure that suspended for a number of minutes the motion of machinery drawn by four horses, furnished ample apology for the amputation of the limb ; nor is it probable that it could have been preserved by any other treatment than that adopted in the case.

We regret that we have not space to continue this subject, for we have many interesting details to furnish in vindication of the claims of the bandage to a much more important place in surgical art than it generally obtains in surgical works. Of its great power over many affections for which it is rarely recommended, we shall present ample proof, and if the clinical reports of Dr. Gibb and Dr. Walker speak strongly in favor of the practice of M. Bauden and Dr. Walker, in comparison with the results of primary amputations, we shall be able to show that the surgical art which understands the bandage, and applies it with judgment, may boast a success and a series of results unattainable by any other means. These are matters of no ordinary interest, and cannot be too frequently enforced upon the attention of the profession. In realizing John Hunter's very correct idea of the difference between true surgical art, and operative surgery, the bandage will maintain a position that bids defiance to rivalry from any quarter. In our next number we shall endeavor to present the subject in its proper light.

